# Protopanaxadiol-Enriched Rice Extracts Suppressed Oxidative and Melanogenic Activities in Melan-a Cells

**DOI:** 10.3390/antiox12010166

**Published:** 2023-01-10

**Authors:** Chaiwat Monmai, Jin-Suk Kim, Karantharat Promyot, So-Hyeon Baek

**Affiliations:** Department of Agricultural Life Science, Sunchon National University, Suncheon 59722, Republic of Korea

**Keywords:** transgenic rice, protopanaxadiol, PPD, antioxidant, anti-melanogenesis, tyrosinase activity, MITF, MAPK pathway, PI3K/Akt pathway

## Abstract

Concerns about hyperpigmentation and skin appearance have led to increasing research into the prevention and altering of skin pigmentation. Natural compounds may be of interest in the search for skin-lightening actives. Protopanaxadiol (PPD), a gut microbiome-induced ginseng metabolite, has been reported to have anti-melanogenic effects. This study aimed to evaluate the antioxidative and anti-melanogenic effects of PPD-enriched rice seed extracts on melan-a cells. The antioxidant and cytotoxicity activities of the extracts were investigated in melan-a cells before measuring their responses to melanogenic activities. The extracts significantly enhanced the antioxidant potency compared with normal rice seed extract. PPD-enriched rice seed extracts (i) significantly downregulated microphthalmia-associated transcription factor, which led to a reduction in tyrosinase and tyrosinase-related protein-1 and -2, (ii) decrease in the cellular tyrosinase activity and melanin content, (iii) reduction in the number of melanin-containing cells, (iv) promotion of melanogenesis downregulators, phosphorylation of extracellular signal-regulated kinase 1/2 and protein kinase B, and (v) downregulation of the phosphorylated p38 mitogen-activated protein kinase and melanin synthesis. These results indicate the feasibility of PPD-enriched rice seed extracts as a novel agent for suppressing melanogenesis and controlling hyperpigmentation.

## 1. Introduction

The skin is the largest body organ and comprises three layers: epidermis, dermis, and subcutis [1]. The epidermis is the outermost layer, and its primary function is to form a barrier against environmental damage [2]. The epidermis is composed mainly of two different cells: The first part is keratinocytes, which are located in the upper layer (constituting approximately 95% of cells), and the second is melanocytes, which are located in the basal layer [3]. Melanin is a pigment and a dominant factor in deciding human skin color [4], which is produced by melanocytes in a process called melanogenesis [5]. The excessive production of melanin results in darker patches on the skin than the normal surrounding skin, which is hyperpigmentation [6]. Thus, cosmetic and pharmaceutical companies are focusing on exploring and repositioning [7] substances that can reduce hyperpigmentation [8] by blocking the melanogenesis pathway [9].

Asian ginseng (*Panax ginseng*) has been widely used in traditional medicine. Ginseng extract (GE), powder, and compounds have been shown to have several pharmacological benefits [10], including antioxidative [11], immunomodulatory [12,13], antitumor [14], and neuroprotective activities [15,16]. Ginsenosides are one of the major biologically active compounds of ginseng [17]. Ginsenosides have two types: protopanaxadiol (PPD; ginsenosides Rb_1_, Rb_2_, Rb_3,_ Rc, Rd, Rg_3_, Rh_2_, etc.) and protopanaxatriol (PPT; ginsenosides Re, Rg_1_, Rg_2_, Rh_1_, etc.) glycosides [18,19]. PPD is the final product of PPD-type saponin that does not exist in natural ginseng and can only be obtained via the intestinal microbial process [20,21,22]. Several reports demonstrated the pharmacological activities of PPD-type ginsenoside in the biological response of skin cells; for example, ginsenoside Rb_3_ decreased the oxidative activities in keratinocytes [23], and ginsenoside Rd enhanced human dermal fibroblast proliferation and collagen synthesis [24], prevention of ginsenoside Rb_1_ on skin photoaging in human dermal fibroblasts [25], and anti-melanogenic activities of ginsenosides Rb1 [26], Rb2 [27], Rg3 [28], and Rh3 [29] in melanocyte cells.

We have developed PPD-enriched rice that can produce PPD from rice seeds directly without the need for an intestinal microbial process [30]. In our previous study, we extracted the transgenic rice seeds, measured the PPD content in the samples, and evaluated the immunomodulatory activities [31]. Our results showed that PPD-enriched transgenic rice seed extracts exhibited positive immunomodulatory potential on RAW264.7 macrophage cells. Additionally, PPD-enriched transgenic rice seed extracts can inhibit osteoclastogenesis by suppressing the nuclear factor of activated T cell 1 expression during osteoclast differentiation [32]. Therefore, this study aimed to evaluate the antioxidative and anti-melanogenic effects of PPD-enriched rice seed extracts on melan-a cells and explore the feasibility of PPD-enriched rice seed extracts as a novel agent for suppressing melanogenesis and controlling hyperpigmentation.

## 2. Materials and Methods

### 2.1. Samples

The PPD-enriched rice seed extracts used in this study were the samples from our previous study [31]. The PPD amount in the rice seed extracts was as follows: DJ (normal rice) = not detectable; #8 = 7.28 ± 0.64 µg/g dry weight (dw) of crude extract; #503 = 1.13 ± 0.03 µg/g dw; #557 = 1.30 ± 0.03 µg/g dw; #564 = 1.28 ± 0.08 µg/g dw; and #595 = 2.36 ± 0.07 µg/g dw (Figures S1 and S2). The GE sample was prepared from a 5-year-old ginseng root and extracted using the same method for the transgenic rice seed extracts [31]. The samples were prepared at concentrations of 10, 25, 50, and 100 mg/mL in dimethyl sulfoxide (DMSO). For the cell treatment, the samples were diluted to a concentration of 10, 25, 50, and 100 µg/mL in cell culture media to maintain the concentration of DMSO at 0.1%. PPD (20S) with a purity of ≥98% HPLC (S-PPD) was obtained from Ambo Institute (Daejeon, Republic of Korea). S-PPD and GE were used as the positive control and prepared at concentrations of 700 ng/mL and 100 mg/mL, respectively.

### 2.2. ABTS Radical Scavenging Ability Assay

**Figure 1:**
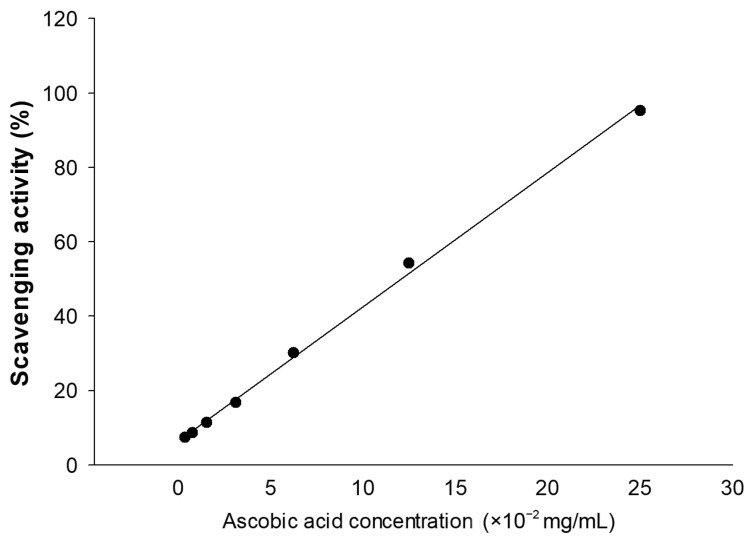
Standard curve of ascorbic acid (vitamin C). The method was linear across a concentration range of 3.9065–250 µg/mL. The regression equation is “y = 3.6145x + 6.3891” (R^2^ = 0.9981); when y is the scavenging activity (%) value.

The antioxidant activity of the PPD-producing transgenic rice seed extracts was determined using the ABTS^+^ cation (ABTS^•+^) decolorization assay method [33] with a slight modification. A mixture of 7 mM ABTS (ROCHE, Basel, Switzerland) and 2.4 mM of potassium persulfate solution (final concentration) was incubated at room temperature and protected from light for 16 h. The working solution of ABTS^•+^ was prepared by diluting the ABTS^•+^ solution with ethanol to an absorbance of 0.70 ± 0.02 at 734 nm. Next, 10 µL of varying concentrations of extracts was introduced to 1 mL ABTS^•+^ working solution. The mixture was incubated in the dark at room temperature. The absorbance at 734 nm was measured after 7 min incubation. The experiment was performed in triplicate, and the combination of 10 µL of distilled water and 1 mL ABTS^•+^ working solution was identified as the control group. Ascorbic acid was used as an antioxidant reference and presented in terms of vitamin C (ascorbic acid) equivalent antioxidant capacity (VCEAC). VCEAC was quantified by constructing a standard curve of ascorbic acid (Figure 1). The percentage of scavenging activity was calculated according to the following formula:

(1) $$\mathrm{Scavenging}\;\mathrm{activity}\;\left(\%\right)=\left[\frac{A_{734}{\;\mathrm{of}\;\mathrm{control}\;-\;A}_{734}\;\mathrm{of}\;\mathrm{sample}\;}{A_{734}\;\mathrm{of}\;\mathrm{control}}\right]\;\times \;100,$$

where A_734_ represents the absorbance at 734 nm.

The concentration of the extracts needed to reduce the initial ABTS^•+^ by 50% (IC_50_) was measured by plotting the percentage of scavenging (*X*) against different concentrations of the extracts (*Y*). The IC_50_ was calculated by substituting the value of *X* with 50 in the regression equation of *Y* = A*X* + B.

### 2.3. Melan-a Cell Culture

Melan-a cells were kindly provided by Professor Sun-Yeou Kim (College of Pharmacy, Gachon University, Republic of Korea) and cultured in a culture medium [RPMI 1640 medium (Gibco™; Thermo Fisher Scientific, Inc., Waltham, MA, USA) containing 10% fetal bovine serum (Gibco™; Thermo Fisher Scientific, Inc., Waltham, MA, USA), 1% penicillin/streptomycin (P/S) (Hyclone Laboratories, Inc., Logan, UT, USA), and 200 nM 12-*O*-tetradecanoylphorbol-13-acetate (Sigma-Aldrich, St. Louis, MO, USA)]. The cells were grown at 37 °C with 5% CO_2_.

### 2.4. Melan-a Cell Viability Assay

The cytotoxicity of the extracts on the melan-a cells was measured using the EZ-CyTox Cell Viability Assay Kit (DoGenBio, Seoul, Republic of Korea). The cells were seeded at 2 × 10^4^ cells/well in a 96-well plate and incubated at 37 °C for 24 h. The culture medium was replaced with the varying concentrations of extracts, and the plate was further incubated for 72 h. The working solution of EZ-CyTox (10-fold dilution in 1× PBS; 110 µL/well) was applied to the cells, and the plate was incubated at 37 °C for 4 h. After incubation, 100 µL of solution from each well was transferred to a new 96-well plate, and the absorbance at 450 nm was recorded using a SpectraMax^®^ ABS Plus Microplate Reader (Molecular Device, San Jose, CA, USA). Culture medium (alone)-treated cells were used as the control group, and the cell viability was assessed using the following formula:

(2) $$\mathrm{Melan-a}\;\mathrm{cell}\;\mathrm{viability}\;\left(\%\right)=\frac{\mathrm{Absorbance}\;\mathrm{at}\;450\;\mathrm{of}\;\mathrm{treatment}}{\mathrm{Absorbance}\;\mathrm{at}\;450\;\mathrm{of}\;\mathrm{control}}\;\times \;100.$$

### 2.5. Melanin Excretion and Melanin Content Assays

Melan-a cells were plated in a 6-well plate at a concentration of 5 × 10^5^ cells/well. After 24 h incubation, 100 µg/mL of DJ, #8, #503, #557, #564, #595, and GE, and 700 pg/mL of S-PPD in the culture media were applied to the cells. The plate was incubated at 37 °C for 72 h, and the melanin excretion was evaluated by the absorbance of the culture medium at 405 nm. The melanin content was measured by disrupting the cell pellets (5 × 10^5^ cells/treatment) with 1 N of NaOH solution at 80 °C for 4 h. The solution was optically evaluated at 405 nm, and the independent experiment was performed in triplicate. The cells treated with the culture media alone were used as the control group.

(3) $$\mathrm{Melanin}\;\mathrm{excretion}\;\mathrm{ratio}\;\left(\%\right)=\frac{\mathrm{Absorbance}\;\mathrm{at}\;405\;\mathrm{nm}\;\mathrm{of}\;\mathrm{treatment}}{\mathrm{Absorbance}\;\mathrm{at}\;405\;\mathrm{nm}\;\mathrm{of}\;\mathrm{control}}\;\times \;100.$$

(4) $$\mathrm{Melanin}\;\mathrm{content}\;\mathrm{ratio}\;\left(\%\right)=\frac{\mathrm{Absorbance}\;\mathrm{at}\;405\;\mathrm{nm}\;\mathrm{of}\;\mathrm{treatment}}{\mathrm{Absorbance}\;\mathrm{at}\;405\;\mathrm{nm}\;\mathrm{of}\;\mathrm{control}}\;\times \;100.$$

### 2.6. L-DOPA Staining Assay

L-DOPA staining was conducted as previously described by Park et al. [34] with a slight modification. Briefly, the treated cells (72 h incubation) were washed with 1× PBS and fixed with 10% formaldehyde for 20 min. The cells were washed with 1× PBS twice and reacted with 2 mg/mL of L-DOPA at 37 °C for 3 h. After washing and drying, the pigmentation of the melan-a cells was observed under an IM-3 series microscope (Optika, Bergamo, Italy).

### 2.7. Cellular Tyrosinase Activity Assay

The cellular tyrosinase activity was measured according to the method by Lee et al. [35]. Melan-a cells (5 × 10^4^ cells/well) were seeded in a 24-well plate and maintained at 37 °C for 24 h. The cells were treated with a non-toxic concentration of treatments and incubated for 72 h. The treated cells were collected using 1× trypsin–EDTA and centrifuged at 2000 rpm at 4 °C for 10 min. The pellets were lysed with 0.1 M sodium phosphate (pH 6.8) containing 1% Triton X-100 on ice for 30 min. The lysates were clarified by centrifugation at 13,000 rpm at 4 °C for 30 min. The supernatant was collected, and the protein concentration was measured using the Bradford method. The volume of an equal amount of protein (40 µg) from each treatment was adjusted with lysis buffer to 80 µL in a 96-well plate. Then, 20 µL of L-DOPA (2 mg/mL: Sigma-Aldrich, St. Louis, MO, USA) was added to each well. The plate was incubated at 37 °C for 1 h, and the solution was optically measured at 475 nm. The culture medium-treated melan-a cells were used as the control group.

(5) $$\mathrm{Cellular}\;\mathrm{tyrosinase}\;\mathrm{activity}\;\left(\%\right)=\left[\frac{A_{475}{\;\mathrm{of}\;\mathrm{control}\;-\;A}_{475}\;\mathrm{of}\;\mathrm{sample}\;}{A_{475}\;\mathrm{of}\;\mathrm{control}}\right]\;\times \;100,$$

where A_475_ represents the absorbance at 475 nm.

### 2.8. Morphological Appearance Using the Fontana–Masson Assay

The effect of the transgenic rice seed extracts on the morphological appearance of the melan-a cells was monitored using the Fontana–Masson staining method. Melan-a cells (5 × 10^4^ cells) were seeded into a 24-well plate and maintained at 37 °C for 24 h. The cells were treated with the indicated concentration of samples and incubated for 72 h. The morphological appearance and melanin pigmentation of the cells were observed using the Fontana–Masson kit (BIOGNOST Ltd., Zagreb, Croatia) following the manufacturer’s instructions. The number of melanin-containing cells was counted by observing 1000 cells under an IM-3 series microscope. A total of 100 melanin-containing cells were distinguished by the morphological appearance into 4 groups (1+, 2+, 3+, and 4+) according to the scoring system described in the report by Rodboon et al. [36].

### 2.9. Quantification of Gene Expression by Real-Time Polymerase Chain Reaction (PCR)

Total RNA was isolated using TRI Reagent™ (Invitrogen, Waltham, MA, USA). The extracted RNA was quantified and qualified using a SpectraMax^®^ ABS Plus Microplate Reader. The ratios of the absorbance at 260 nm and 280 nm (A260:A280) and the absorbance at 260 nm and 230 nm (A260:A230) of the extracted RNA were in the acceptable range (1.8–2.0). First-strand cDNA was synthesized from 1 µg of RNA using the Power cDNA Synthesis Kit (Intron Biotechnology, Seongnam-si, Republic of Korea). The transcripts of the melanogenesis-related genes were monitored using a CFX Connect Real-Time PCR system (Bio-Rad, Hercules, CA, USA). The qPCR reaction consisted of 5 ng of cDNA template and 0.375 µM of each primer (forward and reverse primer) in a total volume of 20 µL of RealMOD™ Green W^2^ 2× qPCR Mix (Intron Biotechnology, Seongnam-si, Republic of Korea). The specific primer sets used in the experiment are shown in Table 1. The PCR condition was conducted according to Monmai et al. [31]. The relative gene expression levels were analyzed using the CFX Connect Real-Time PCR program with regard to glyceraldehyde 3-phosphate dehydrogenase (GAPDH).

### 2.10. Western Blot Assay

The treated cells were collected in a 1.5 mL tube and disturbed with radioimmunoprecipitation assay buffer (GeneAll Biotechnology, Seoul, Republic of Korea) containing 1× Protease Inhibitor Cocktail Kit 5 (Bio-Medical Science Co., Ltd., Seoul, Republic of Korea) on ice for 30 min. The tubes were centrifuged at 13,000 rpm at 4 °C for 30 min. The protein solution was collected in a new 1.5 mL tube. The protein concentrations were measured using the Bradford reagent (Sigma-Aldrich, St. Louis, MO, USA) and quantified by comparison with a bovine serum albumin standard curve. The protein (30 µg) was fractionated with sodium dodecyl sulfate-polyacrylamide gel electrophoresis and transferred onto a nitrocellulose membrane. The transferred protein was incubated with antibodies specific to the melanogenesis-related proteins (microphthalmia-associated transcription factor (MITF), tyrosinase-related protein-1 (TRP-1), TRP-2, and tyrosinase) (Santa Cruz Biotechnology, Dallas, TX, USA), mitogen-activated protein kinases (MAPKs: p38 MAPK, phosphorylation (p)-p38 MAPK, extracellular signal-regulated kinase (ERK), and p-ERK) (Cell Signaling, Danvers, MA, USA), and phosphatidylinositol 3-kinase (PI3K)/protein kinase B (Akt and p-Akt; Cell Signaling, Danvers, MA, USA) signaling pathways. Glyceraldehyde 3-phosphate dehydrogenase (GAPDH; Santa Cruz Biotechnology, Dallas, TX, USA) was used as the protein loading control. The signaling of specific proteins was detected using Clarity™ Western ECL Substrate (Bio-Rad, Hercules, CA, USA). A ChemiDoc Imaging System (Bio-Rad, Hercules, CA, USA) was used to image and quantify the protein signaling in terms of intensity.

### 2.11. Statistical Analysis

The data are shown as means ± standard deviations. The statistical analyses were performed using Statistix (version 8.1; Statistix, Tallahassee, FL, USA). The data analysis was performed using a one-way analysis of variance followed by post-hoc Duncan’s multiple range tests. The differences between the two groups were assessed using *t*-tests at a significance level of *p* < 0.05.

## 3. Results

### 3.1. Effects of Transgenic Rice Seed Extracts on Antioxidant Activity

The antioxidant effects of the PPD-enriched transgenic rice seed extracts and normal rice seed extract (DJ) were evaluated using the ABTS radical scavenging method and were compared with that of commercially synthesized PPD (S-PPD) and natural GE. The antioxidant effect of various concentrations (10, 25, 50, and 100 mg/mL) of transgenic and DJ rice seed extracts is shown in Table 2. In each treatment, the antioxidant activity increased with the increase in the treatment concentration. The comparison of the antioxidant activity of the transgenic rice seed extracts at the same concentration showed that treatment with #8 significantly promoted the antioxidant activity by providing the highest level of ABTS radical scavenging. However, no significant difference in antioxidant activity (*p* < 0.05) was observed among #8 (100 mg/mL), GE (100 mg/mL), and S-PPD (700 ng/mL).

Additionally, the antioxidant activity was also presented in terms of VCEAC, which was quantified by constructing a standard curve of ascorbic acid at the concentration range of 3.9065–250 µg/mL (0.39065 × 10^−2^–25 × 10^−2^ mg/mL; R^2^ = 0.9981). Moreover, the antioxidant activity of the transgenic rice seed extracts (ABTS radical scavenging (%) and VCEAC) was significantly correlated with their PPD content (Pearson’s correlation coefficient = 0.745; the critical value for Pearson’s *r* at the degree of freedom at 18 and *p* < 0.01 = 0.561). A lower IC_50_ specified a higher antioxidant activity. Sample #8 exhibited the highest antioxidant activity among the PPD-enriched rice seed extracts (Table 2).

### 3.2. Effects of Transgenic Rice Seed Extracts on Melan-a Cell Viability

**Figure 2:**
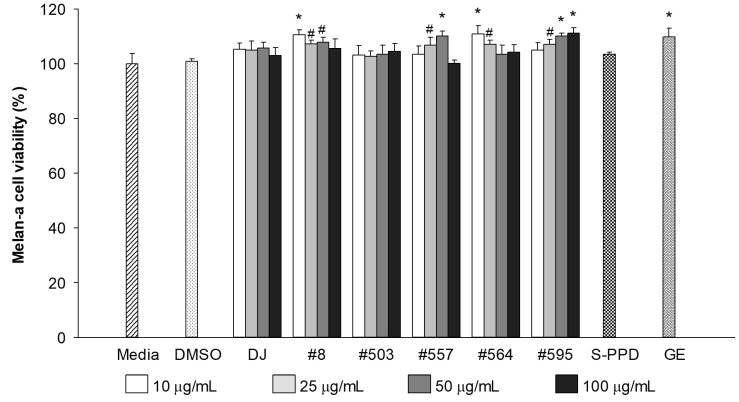
Effects of the PPD-enriched transgenic rice seed extracts on melan-a cell viability. DMSO, S-PPD, and GE concentrations were 0.1%, 700 pg/mL, and 100 µg/mL, respectively. Data are shown as mean ± standard deviation (*n* = 3). Significant differences at *p* < 0.05 (#) and *p* < 0.01 (*) were determined via comparisons with the media group.

The cytotoxicity of the PPD-enriched transgenic rice seed extracts was evaluated at a final concentration of 10–100 µg/mL. The results showed that treatment with DJ (normal rice) and transgenic rice seed extracts up to 100 µg/mL did not show any toxicity in melan-a cells (Figure 2). Similar to the GE- (100 µg/mL) and S-PPD- (700 pg/mL) treated cells, there was no significant difference in melan-a cell viability compared with the untreated cells (the media group). Based on the antioxidant (Table 2) and melan-a cell viability (Figure 2), the treatment concentration of 100 mg/mL (100 µg/mL final concentration) was selected for further experiments.

### 3.3. Effects of Transgenic Rice Seed Extracts on Melanin Content and Melanin Excretion

**Figure 3:**
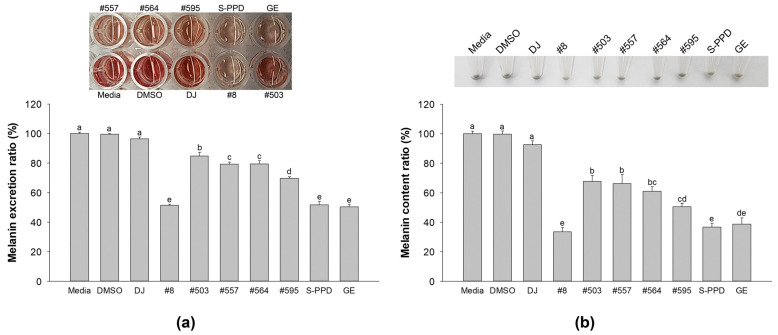
Effects of the PPD-enriched transgenic rice seed extracts on melanin excretion and melanin content. Effects on (**a**) melanin excretion and (**b**) melanin content. DJ, #8, #503, #557, #564, and #595 concentrations were 100 µg/mL. DMSO, S-PPD, and GE concentrations were 0.1%, 700 pg/mL, and 100 µg/mL, respectively. Data are shown as mean ± standard deviation (*n* = 3). Lowercase letters indicate significant differences at *p* < 0.05 among the treatments.

The culture medium was transferred to a new 96-well plate and optically measured at 405 nm to evaluate the melanin excretion activity. Figure 3a shows that the transgenic rice seed extracts significantly reduced melanin excretion compared with DJ (normal rice seed extract) (*p* < 0.05). Among the transgenic rice seed extracts, the highest decreasing activity of melanin excretion was observed in the #8-treated cells. Then, the treated cells were collected and counted for 5 × 10^5^ cells, and the melanin content was investigated. The results showed that the pellets of the DJ-treated cells were more deeply dark (eye observation) than those observed in the transgenic rice seed extract-treated cells (Figure 3b). This observation was confirmed by the cellular melanin content result. The PPD-enriched transgenic rice seed extracts significantly inhibited the cellular melanin compared with the DJ group (Figure 3b). No significant differences in melanin excretion and melanin content were observed among the #8- (the highest PPD content), S-PPD-, and GE-treated cells.

### 3.4. Effects of Transgenic Rice Seed Extracts on the Cellular Tyrosinase Activity

**Figure 4:**
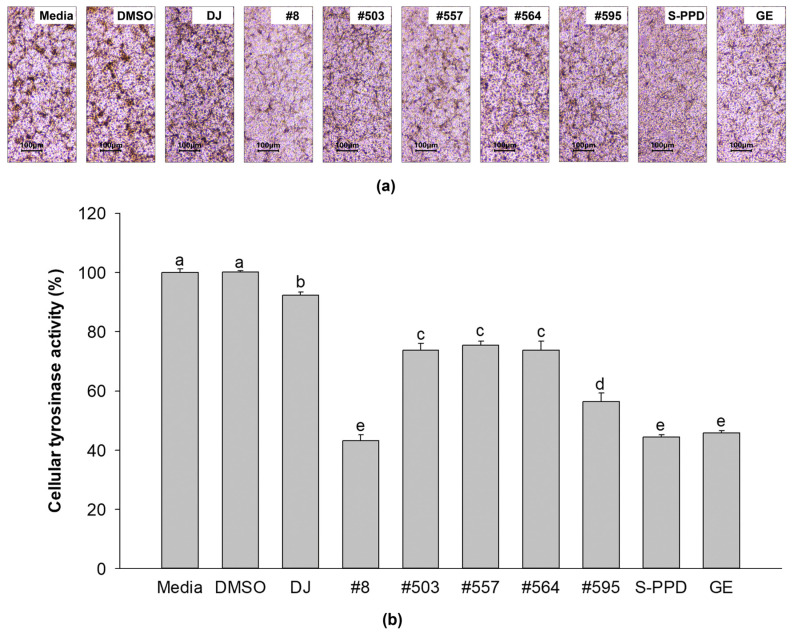
Effects of the PPD-enriched transgenic rice seed extracts on cellular tyrosinase activity. (**a**) L-DOPA staining in melan-a cells and (**b**) cellular tyrosinase activity. DJ, #8, #503, #557, #564, and #595 concentrations were 100 µg/mL. DMSO, S-PPD, and GE concentrations were 0.1%, 700 pg/mL, and 100 µg/mL, respectively. Data are shown as mean ± standard deviation (*n* = 3). Lowercase letters indicate significant differences at *p* < 0.05 among the treatments.

Medium- and DMSO-treated cells showed that most of the cells exhibited dark spots (Figure 4a). The cells from the transgenic rice seed extract groups showed lighter dark spots than the normal cells (the media group). Treatment with #8 (the highest PPD content) significantly reduced the dark spots compared with the other transgenic rice seed extracts. An equal amount of cellular protein from each treatment was incubated with L-DOPA and optically measured to confirm the tyrosinase activity of the transgenic rice seed extracts. As shown in Figure 4b, the transgenic rice seed extracts significantly decreased the cellular tyrosinase activity compared with the media, DMSO, and DJ (normal rice) groups. Treatment of the cells with #8 exhibited the lowest tyrosinase activity among all transgenic rice seed extracts (43.11 ± 2.02%). No significant difference in cellular tyrosinase activity was observed among the #8- (the highest PPD content), S-PPD-, and GE-treated cells.

### 3.5. Effects of Transgenic Rice Seed Extracts on Melanin-Containing Melan-a Cells

**Figure 5:**
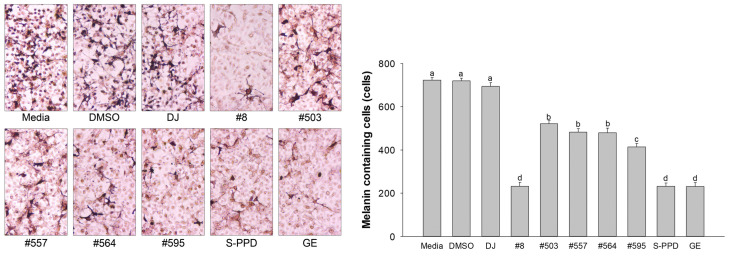
Effects of the PPD-enriched transgenic rice seed extracts on melanin-containing melan-a cells. Melanin-containing melan-a cells were counted by observing 1000 cells under a microscope. DJ, #8, #503, #557, #564, and #595 concentrations were 100 µg/mL. DMSO, S-PPD, and GE concentrations were 0.1%, 700 pg/mL, and 100 µg/mL, respectively. Data are shown as mean ± standard deviation (*n* = 3). Lowercase letters indicate significant differences at *p* < 0.05 among the treatments.

The melanin-containing melan-a cells were evaluated using the Fontana–Masson staining assay. The melanin pigment was stained and shown as dark spots. Treatment with the transgenic rice seed extracts significantly decreased the number of melanin-containing cells (232 ± 18.3–522 ± 13.7 cells/1000 cells) compared with the DJ (normal rice) group (694 ± 18.0 cells/1000 cells) (Figure 5). Additionally, treatment with the transgenic rice seed extracts reduced the dark pigmentation by eye observation under a microscope. Treatment with #8 (the highest PPD content), S-PPD, and GE showed the lowest number of melanin-containing melan-a cells at 232 ± 18.3, 231 ± 16.0, and 231 ± 17.5 cells/1000 cells, respectively.

### 3.6. Effects of Transgenic Rice Seed Extracts on the Morphological Appearance of Melan-a Cells

**Figure 6:**
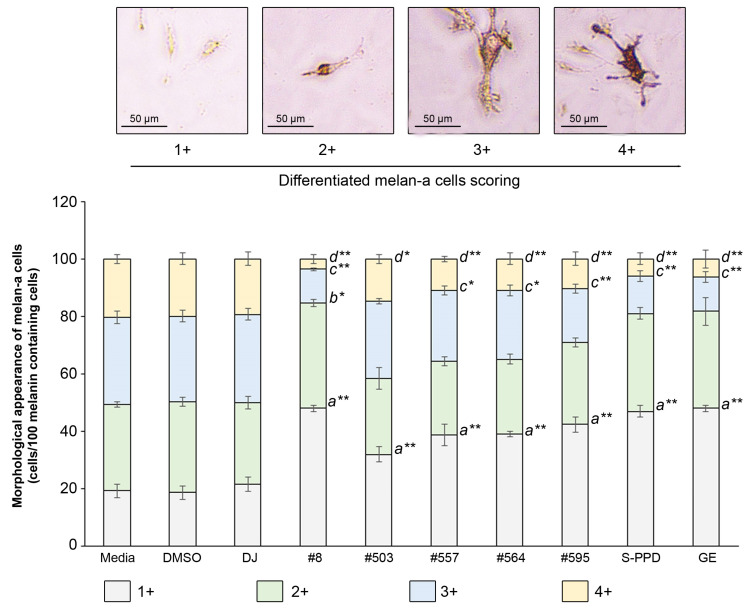
Effects of the PPD-enriched transgenic rice seed extracts on the morphological appearance of melan-a cells. The melanin-containing cells were 100 cells and divided into 4 groups based on their morphology. DJ, #8, #503, #557, #564, and #595 concentrations were 100 µg/mL. DMSO, S-PPD, and GE concentrations were 0.1%, 700 pg/mL, and 100 µg/mL, respectively. Data are shown as mean ± standard deviation (*n* = 3). Lowercase letters indicate significant differences at *p* < 0.05 (*) and *p* < 0.01 (**) versus scores 1+, 2+, 3+, and 4+ of the media group.

A total of 100 melanin-containing cells were counted and divided into four groups according to the differentiation score (1+, 2+, 3+, and 4+) (Figure 6). Treatment with the PPD-enriched transgenic rice seed extracts significantly reduced the population of 4+ differentiated melan-a cells and increased the 1+ differentiated melan-a cells. For treatment with #8 (the highest PPD content), the number of 1+ differentiated cells markedly increased (*p* < 0.01) from 19 ± 2.31 cells (media group) to 48 ± 1.00 cells, and the population of 2+ differentiated cells increased (*p* < 0.05) from 30 ± 1.00 cells (media group) to 37 ± 1.15 cells. The number of 3+ and 4+ differentiated cells of #8 significantly reduced (*p* < 0.01) to 12 ± 0.58 and 4 ± 1.53 cells, respectively, compared with the media group (30 ± 2.08 and 20 ± 1.53 cells, respectively).

### 3.7. Effects of Transgenic Rice Seed Extracts on Melanogenic-Related Gene Expression

**Figure 7:**
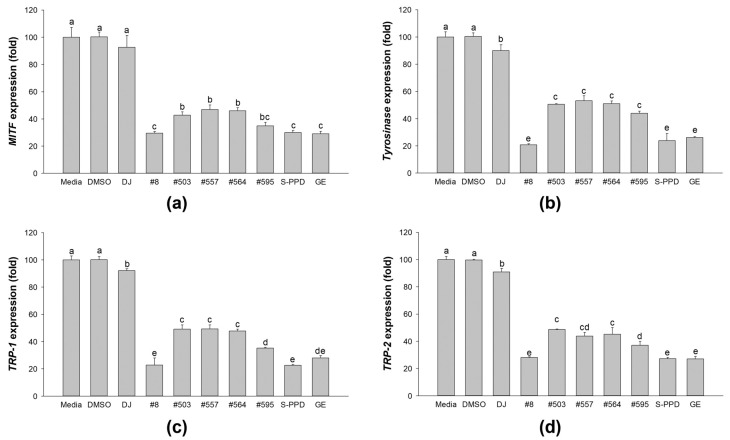
Effects of the PPD-enriched transgenic rice seed extracts on mRNA expression levels of melanogenesis mediators in melan-a cells. The expression levels of (**a**) MITF, (**b**) tyrosinase, (**c**) TRP-1, and (**d**) TRP-2. DJ, #8, #503, #557, #564, and #595 concentrations were 100 µg/mL. DMSO, S-PPD, and GE concentrations were 0.1%, 700 pg/mL, and 100 µg/mL, respectively. Data are shown as mean ± standard deviation (*n* = 3). Lowercase letters indicate significant differences at *p* < 0.05 among the treatments.

The gene expression levels of MITF, tyrosinase, TRP-1, and TRP-2 were measured to confirm the melanogenesis inhibition activity of the transgenic rice seed extracts (Figure 7). The results demonstrated that treatment with the PPD-enriched rice seed extracts gradually suppressed the expression levels of MITF, tyrosinase, TRP-1, and TRP-2 in melan-a cells. Moreover, treatment with #8 (the highest PPD content) showed markedly lower expression levels of MITF, tyrosinase, TRP-1, and TRP-2, which is similar to the results of the S-PPD- and GE-treated cells.

### 3.8. Effects of Transgenic Rice Seed Extracts on Melanogenesis-Related Proteins

**Figure 8:**
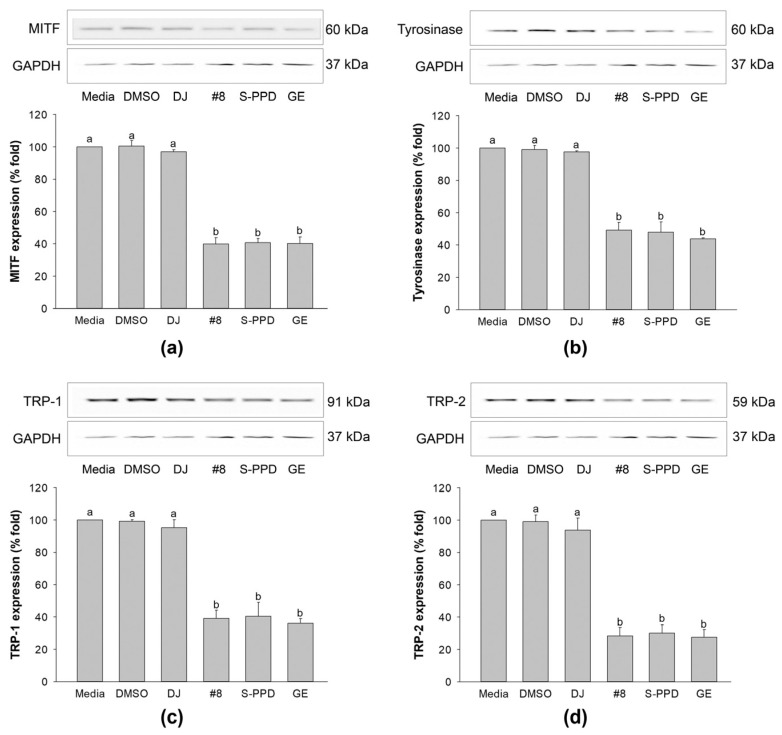
Effects of the transgenic rice seed extracts on melanogenesis-related proteins. Effects on (**a**) MITF, (**b**) tyrosinase, (**c**) TRP-1, and (**d**) TRP-2 expression levels. DJ, #8, and GE concentrations were 100 µg/mL. S-PPD and DMSO concentrations were 700 pg/mL and 0.1%, respectively. Data are shown as means ± standard deviations. Lowercase letters indicate significant differences among treatments at *p* < 0.05.

The above results indicated that treatment with #8 (highest PPD content) caused higher anti-melanogenic effects than those exerted by the other transgenic rice lines. Therefore, #8 was selected to evaluate the molecular mechanism of PPD-enriched rice on the expression levels of MITF, tyrosinase, TRP-1, and TRP-2. The MITF, a transcription factor that is involved in melanogenesis and is a regulator of TRP-1, TRP-2, and tyrosinase [37], exhibited a high expression level in the cells treated with media, 0.1% DMSO, and 100 µg/mL of DJ (Figure 8a). Treatment with #8, S-PPD, and GE, containing PPD, significantly decreased the expression level of this transcription factor compared with DJ (normal rice). The reduction in the MITF level decreased tyrosinase, TRP-1, and TRP-2 in melan-a cells (Figure 8b–d). In treatment with #8, no significant difference was observed in the expression level of these proteins compared with the S-PPD and GE groups (*p* < 0.05).

### 3.9. Effects of Transgenic Rice Seed Extracts on the MAPKs (ERK 1/2 and p38) and PI3K/Akt Signaling Pathways

**Figure 9:**
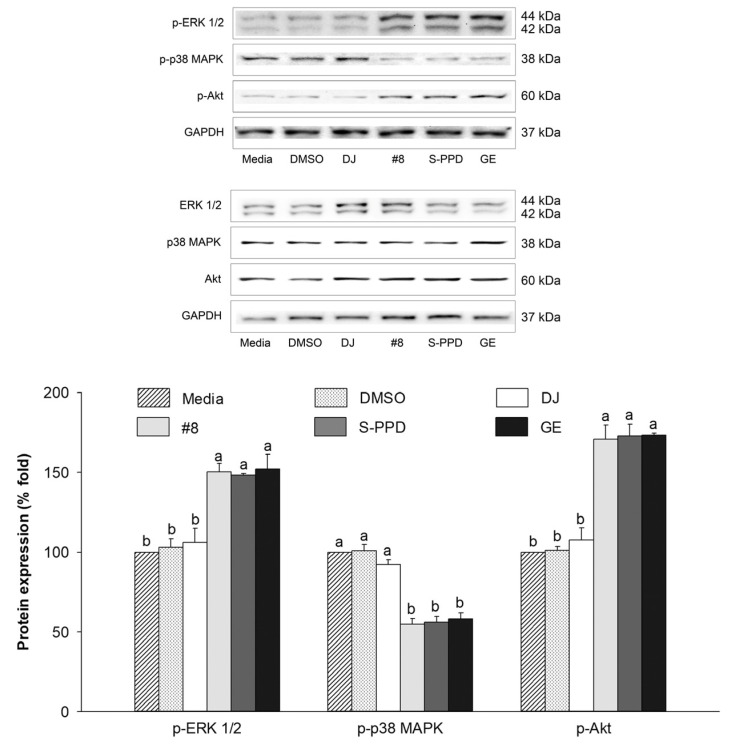
Effects of the transgenic rice seed extracts on the MAPKs (ERK 1/2 and p38) and PI3K/Akt signaling pathways. DJ, #8, and GE concentrations were 100 µg/mL. S-PPD and DMSO concentrations were 700 pg/mL and 0.1%, respectively. Data are shown as means ± standard deviations. Lowercase letters indicate significant differences among treatments at *p* < 0.05.

The regulation of MITF is related to the PI3K/Akt and MAPK signaling pathways [38]. The effect of the transgenic rice seed extracts on the protein expression levels of p-ERK 1/2, p-p38, and p-Akt was evaluated. Compared with the media group, the expression levels of these proteins were reduced in the #8-, S-PPD-, and GE-treated cells (Figure 9). However, treatment with 0.1% DMSO and 100 µg/mL of DJ (normal rice) showed the same levels (*p* < 0.05) of these proteins compared with the media group. For the PPD-containing groups, no significant difference in these protein levels was observed in the #8-, S-PPD-, and GE-treated cells (*p* < 0.05).

## 4. Discussion

In this study, the PPD-enriched transgenic rice seed extracts significantly enhanced the antioxidant effect compared with the normal rice seed extract (DJ) by showing a higher percentage of ABTS radical scavenging and VCEAC (Table 2). The highest antioxidative activities were seen in #8 (100 mg/mL), which showed similar results to those of the S-PPD (700 ng/mL) and GE groups (100 mg/mL). Additionally, the antioxidant activity was related to the amount of PPD in the transgenic rice seed extracts (Pearson’s correlation coefficient at *p* < 0.01). Deng et al. [39] reported that the antioxidant activity correlated with the PPD due to its ability to scavenge toxic free radicals. Additionally, the mixture of PPD and PPT at the ratio of 1:1 also showed an increasing antioxidative effect in HepG2 [40].

**Figure 10:**
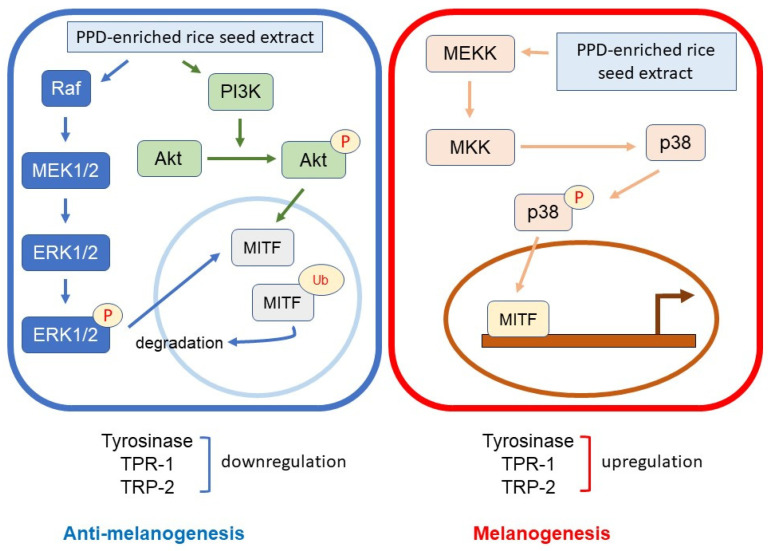
Schematic diagram of the activation of p-ERK 1/2, p-Akt, and p-p38 on melanogenesis regulation.

Melanin synthesis is controlled by the regulatory mechanism in the melanogenesis pathway [41]. MITF plays an important role as a melanogenesis regulator [42], and the activation of MITF causes upregulation of the key melanogenesis regulation enzymes, such as tyrosinase, TRP-1, and TRP-2 [43]. Melanogenesis is activated by several endogenous and exogenous stimulants, such as ultraviolet radiation, metabolic disorders, estrogen, and skin irritation [44]. In addition to those stimulants, the activation of the MAPK and PI3K/Akt signaling pathways has been reported to play an essential role in melanogenesis [44,45]. The MITF ubiquitination and degradation were enhanced by p-ERK and p-Akt, which caused a decrease in tyrosinase expression and tyrosinase activity [46,47] (Figure 10). As the rate-limiting enzyme is involved in melanogenesis, reducing tyrosinase leads to the suppression of melanogenesis [48]. Conversely, the activation of p38 MAPK (p-p38 MAPK) could promote melanogenesis by inhibiting the degradation of MITF [49,50]. During melanogenesis, tyrosinase plays an important role in converting tyrosine to L-DOPA and L-DOPA to dopachrome to form melanin [6,51]. Therefore, the reduction in tyrosinase activity and melanogenic-involved elements (MITF, TRP-1, and TRP-2) played a critical role in developing the anti-pigmentation agents. This study showed that treatment with the transgenic rice seed extracts containing PPD significantly downregulated MITF expression at the mRNA and protein levels (Figure 7 and Figure 8). Additionally, the PPD-enriched transgenic rice seed extracts significantly promoted the expression level of p-ERK 1/2 and p-Akt (MITF degradation inducers) and suppressed the p-p38 MAPK expression (melanogenesis enhancer) (Figure 9). The regulation of p-p38 MAPKs, p-ERK 1/2, and p-Akt may affect the inhibition of melanogenesis. The expression levels of tyrosinase, TRP-1, and TRP-2 (both mRNA and protein levels) significantly decreased due to the reduction in MITF. The reduction of tyrosinase in the PPD-transgenic rice seed extract-treated cells was also confirmed using L-DOPA (tyrosinase substrate in melanin synthesis). The results showed that the PPD-transgenic rice seed extracts markedly suppressed the tyrosinase activity (Figure 4). In addition to the inhibition effect on tyrosinase-related melanogenesis, the PPD-transgenic rice seed extracts also affected the melan-a cell morphological appearance by decreasing the number of melanin-containing cells as well as the cell size and cellular melanin distribution (Figure 5 and Figure 6). Furthermore, the extracts reduced cell differentiation-related melanin excretion and melanin accumulation.

## 5. Conclusions

This study demonstrated the antioxidative and anti-melanogenic potential of PPD-enriched rice seed extracts as a new source of melanogenesis and hyperpigmentation controllers. PPD-enriched rice seed extracts suppressed the tyrosinase, TRP-1, and TRP-2 expressions at the mRNA and protein levels and reduced melanin content by downregulating MITF via interference with p-ERK 1/2 and p-Akt. Furthermore, the differentiation of melanin-enriched cells interfered with PPD-enriched rice seed extracts, which led to the reduction in cell size and melanin contribution in melan-a cells. These findings showed that PPD-enriched rice seeds that do not require complex intestinal microbial processes might be considered a potentially useful natural depigmentation agent.

## Figures and Tables

**Table 1:** Nucleotide sequences of the primers used in this experiment.

Gene	Accession Number	Sequence (5′-3′)	Target Size (bp)
*MITF*	NM_001113198.2	Forward primer: AGCGTGTATTTTCCCCACAG	239
		Reverse primer: CCTTAGCTCGTTGCTGTTCC	
*TRP-1*	NM_031202.3	Forward primer: TCTGGCCTCCAGTTACCAAC	223
		Reverse primer: TCAGTGAGGAGAGGCTGGTT	
*TRP-2*	X63349.1	Forward primer: ACCCTGTGTTTGTGGTCCTC	186
		Reverse primer: GTTGCTCTGCGGTTAGGAAG	
*Tyrosinase*	D00131.1	Forward primer: CCAGAAGCCAATGCACCTAT	193
		Reverse primer: CCAGATACGACTGGCCTTGT	
*GAPDH*	NM_001289726.2	Forward primer: AACTTTGGCATTGTGGAAGG	223
		Reverse primer: ACACATTGGGGGTAGGAACA	

**Table 2:** Antioxidant activity of the PPD-enriched transgenic rice seed extracts.

Treatment	Concentration (mg/mL)	Antioxidant Activity		IC_50_ (mg/mL)
		ABTS Radical Scavenging (%)	Vitamin C Equivalent Antioxidant Capacity (VCEAC: mg/g Dry Weight)	
DMSO	0.1%	0.08 ± 0.11	−	−
DJ	10	8.09 ± 1.20 ^d^	0.005 ± 0.003 ^d^	Not in the range (<50%)
	25	11.86 ± 0.65 ^d^	0.015 ± 0.002 ^d^	
	50	23.81 ± 0.98 ^d^	0.048 ± 0.003 ^d^	
	100	39.83 ± 1.63 ^d^	0.093 ± 0.005 ^d^	
#8	10	25.81 ± 0.98 ^a^	0.054 ± 0.003 ^a^	37.90 ± 0.30
	25	42.53 ± 1.09 ^a^	0.100 ± 0.003 ^a^	
	50	60.94 ± 0.76 ^a^	0.151 ± 0.002 ^a^	
	100	96.46 ± 1.31 ^a^	0.249 ± 0.004 ^a^	
#503	10	15.02 ± 3.16 ^c^	0.024 ± 0.009 ^c^	64.27 ± 0.15
	25	25.35 ± 0.98 ^c^	0.052 ± 0.003 ^c^	
	50	42.91 ± 1.20 ^c^	0.101 ± 0.003 ^c^	
	100	71.49 ± 1.31 ^c^	0.180 ± 0.004 ^c^	
#557	10	16.10 ± 1.42 ^bc^	0.027 ± 0.004 ^bc^	60.41 ± 2.17
	25	26.96 ± 1.09 ^c^	0.057 ± 0.003 ^c^	
	50	44.99 ± 1.31 ^c^	0.107 ± 0.004 ^c^	
	100	74.96 ± 1.63 ^c^	0.190 ± 0.005 ^c^	
#564	10	16.02 ± 0.87 ^bc^	0.027 ± 0.002 ^bc^	60.50 ± 1.22
	25	27.27 ± 1.31 ^c^	0.058 ± 0.004 ^c^	
	50	44.14 ± 0.33 ^c^	0.104 ± 0.001 ^c^	
	100	75.27 ± 2.51 ^bc^	0.191 ± 0.007 ^bc^	
#595	10	21.57 ± 1.74 ^ab^	0.042 ± 0.005 ^ab^	50.73 ± 0.72
	25	33.05 ± 2.07 ^b^	0.074 ± 0.006 ^b^	
	50	51.23 ± 0.98 ^b^	0.124 ± 0.003 ^b^	
	100	82.13 ± 2.83 ^b^	0.210 ± 0.008 ^b^	
S-PPD	70 ng/mL	21.73 ± 0.87 ^ab^	0.042 ± 0.002 ^ab^	275.82 ± 5.35 ng/mL
	175 ng/mL	42.60 ± 2.07 ^a^	0.100 ± 0.006 ^a^	
	350 ng/mL	62.87 ± 0.87 ^a^	0.156 ± 0.002 ^a^	
	700 ng/mL	94.61 ± 0.65 ^a^	0.244 ± 0.002 ^a^	
GE	10 mg/mL	26.43 ± 0.33 ^a^	0.055 ± 0.001 ^a^	38.26 ± 0.63
	25 mg/mL	42.06 ± 0.44 ^a^	0.099 ± 0.001 ^a^	
	50 mg/mL	59.40 ± 1.20 ^a^	0.147 ± 0.003 ^a^	
	100 mg/mL	94.07 ± 1.42 ^a^	0.243 ± 0.004 ^a^	

Data are shown as mean ± standard deviation (*n* = 3). Lowercase letters indicate significant differences at *p* < 0.05 among treatments at the same concentration.

## Data Availability

Data is contained within the article and supplementary material.

## Supplementary Materials

The following supporting information can be downloaded at: https://www.mdpi.com/article/10.3390/antiox12010166/s1, Figure S1: The calibration curve of PPD over the concentration range of 0.0156–0.50000 ppm; Figure S2: PPD production in the transgenic rice seed extracts.
