# Supplementary material for: Protopanaxadiol-Enriched Rice Extracts Suppressed Oxidative and Melanogenic Activities in Melan-a Cells
Source: Antioxidants (Basel). 2023 Jan 10;12(1):166. doi: 10.3390/antiox12010166 (PMC9854995; doi:10.3390/antiox12010166)

Supplementary Materials

Figure S1. The calibration curve of PPD over the concentration range of 0.0156–0.50000 ppm.

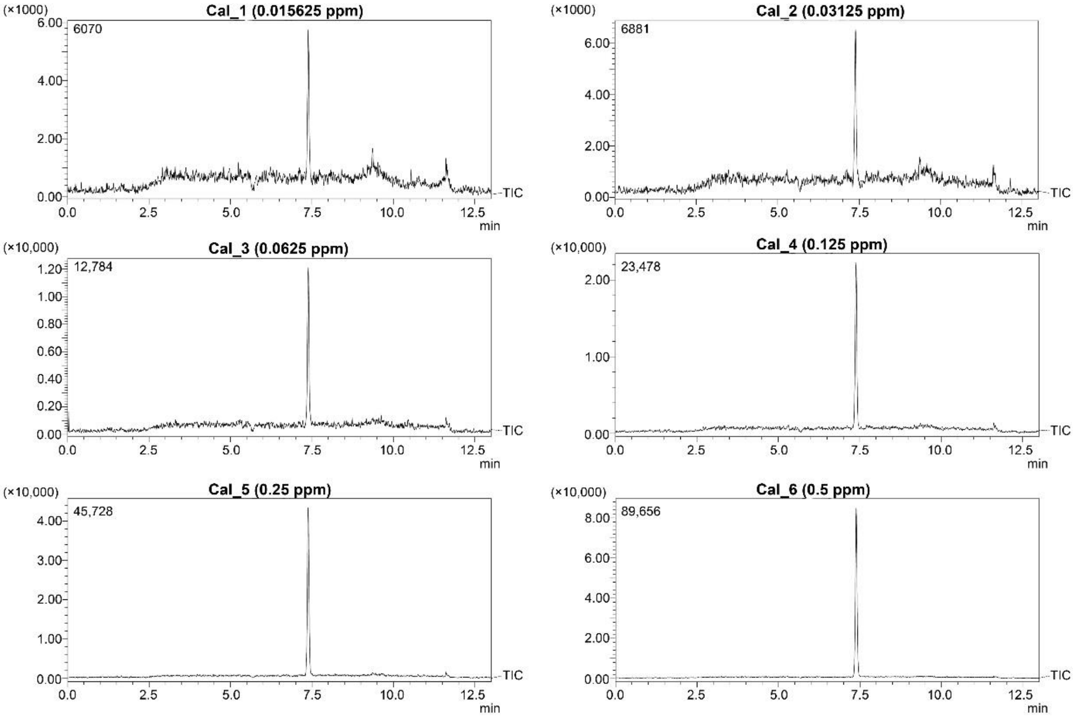

Calibration curve

ID#	: 1	m/z	: 425.0000>407.2000
Name	: PPD		
Quantitative method	: External standard		
Function	: f(x) = 202,985x + 2592.25		

Rr1 = 0.9992163 Rr2 = 0.9984331

MeanRF: 2.644939×10^5 RF SD: 9.865022×10^4 RF %RSD: 37.297735

FitType	: Linear
ZeroThrough	: Not through
Weighted regression	: None

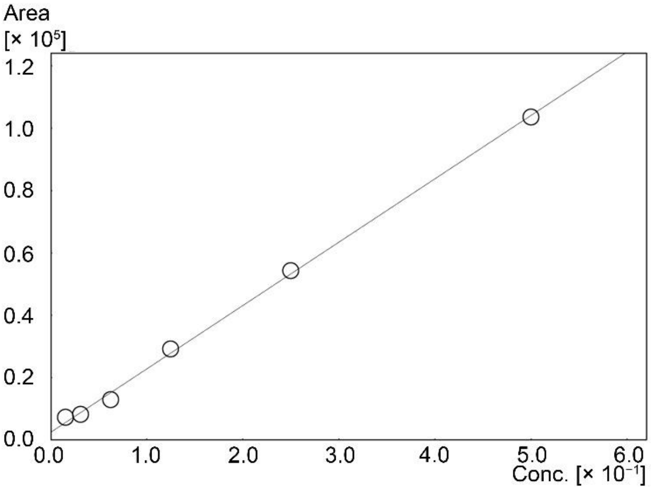

#	Conc. (Ratio)	MeanArea	Area
1	0.015625	7210	7210
2	0.03125	8164	8164
3	0.0625	12,890	12,890
4	0.125	29,202	29,202
5	0.25	54,308	54,308
6	0.5	103,592	103,592

Figure S2. PPD production in the transgenic rice seed extracts. (a) Representation of the MS chromatograms of PPD detected in transgenic rice seed extracts (the Y axis of sample #8 is ×100,000, whereas the Y axes of the other samples are ×10,000). (b) The amount of PPD in each transgenic rice line. Data are presented as means ± standard deviations. Lowercase letters (a, b, c, and d) indicate significant differences at $p < 0.05$ among the treatments.

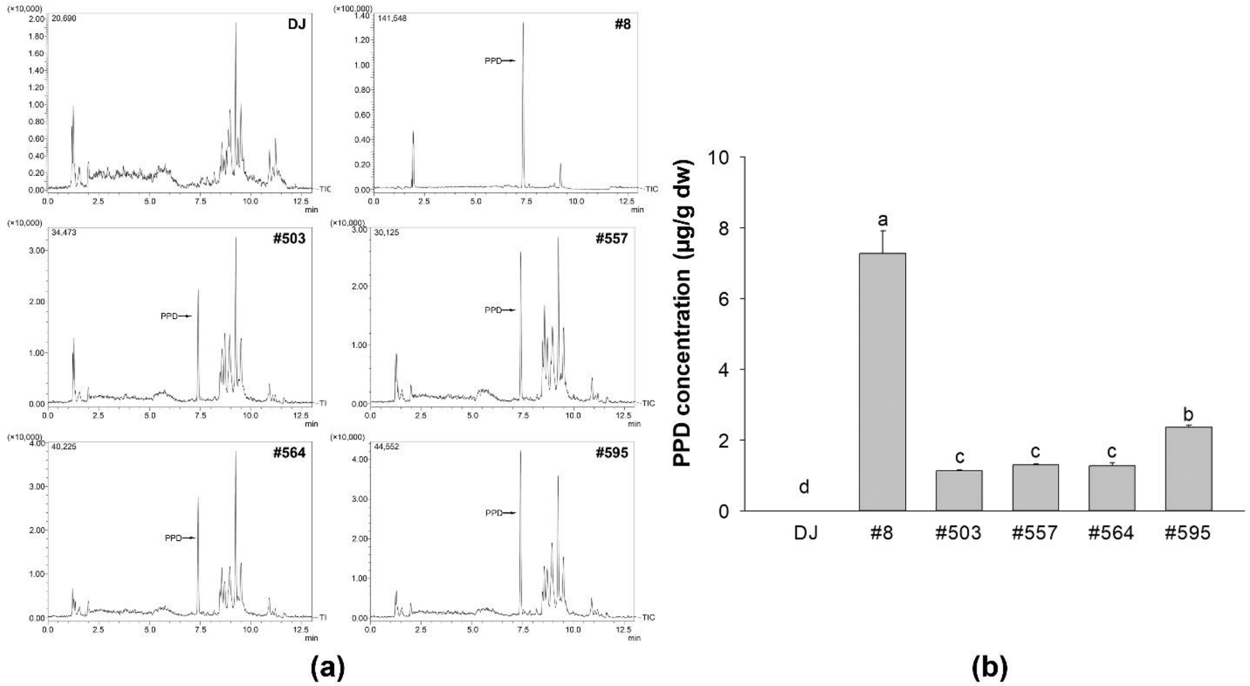

Supplement: Supplementary file 1 [file antioxidants-12-00166-s001.zip › Supplementary Materials.pdf]
