# Supplementary figures and images for: Protopanaxadiol-Enriched Rice Extracts Suppressed Oxidative and Melanogenic Activities in Melan-a Cells
Source: Antioxidants (Basel). 2023 Jan 10;12(1):166. doi: 10.3390/antiox12010166 (PMC9854995; doi:10.3390/antiox12010166)

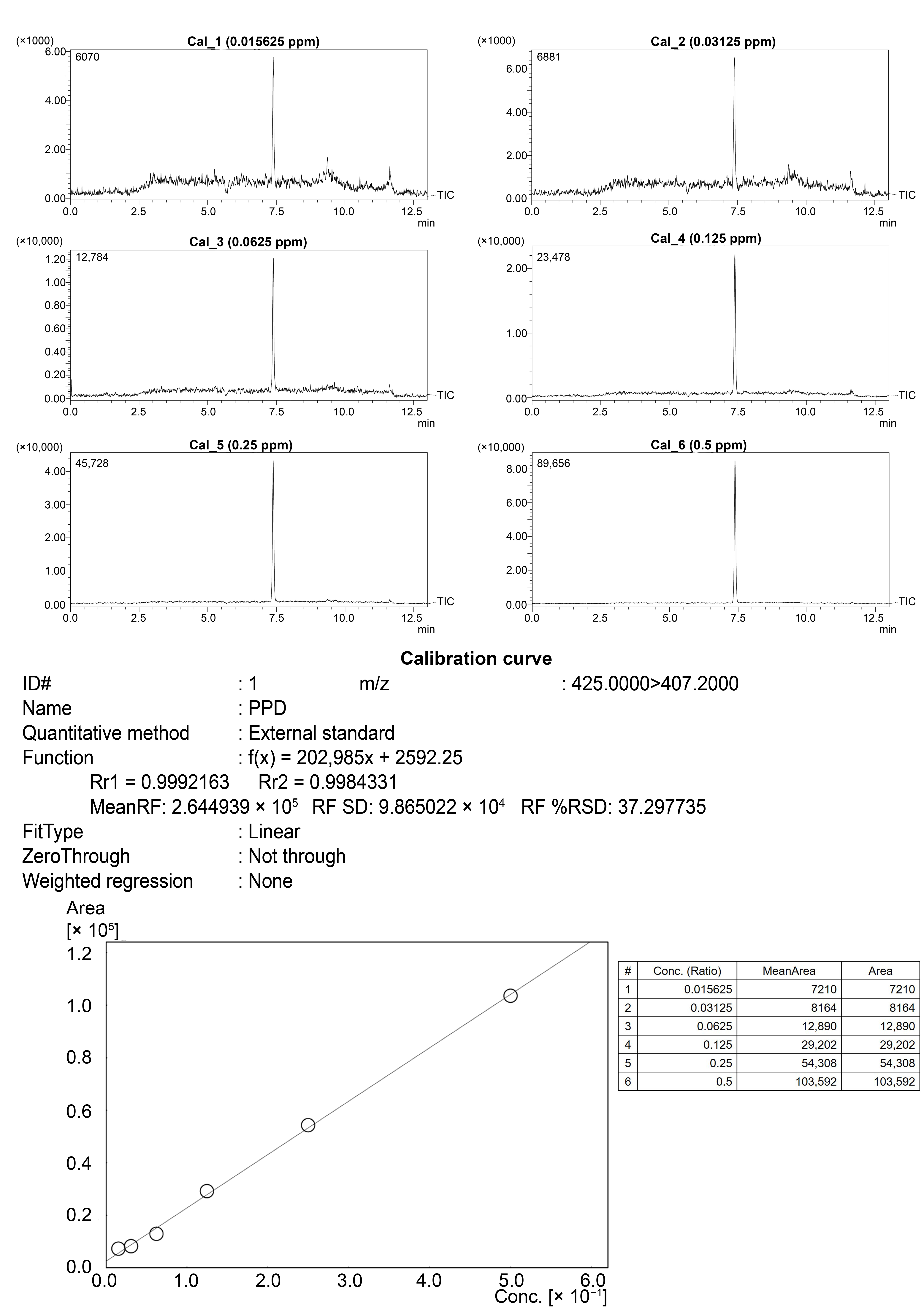

Supplement: Supplementary file 1 [file antioxidants-12-00166-s001.zip › Figure S1.jpg]

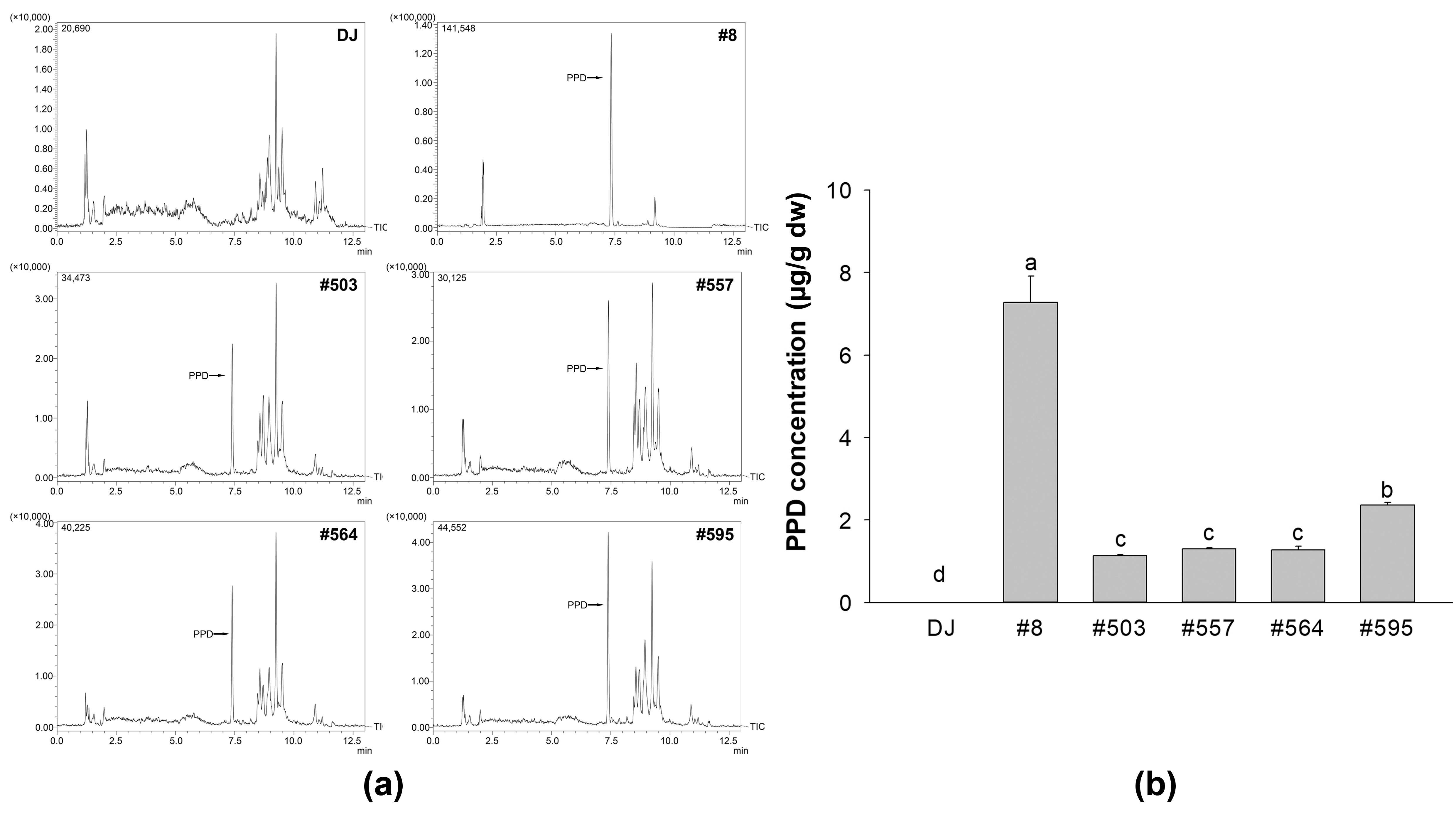

Supplement: Supplementary file 1 [file antioxidants-12-00166-s001.zip › Figure S2.jpg]
